# Altered Expression of Candidate Genes in Mayer–Rokitansky–Küster–Hauser Syndrome May Influence Vaginal Keratinocytes Biology: A Focus on Protein Kinase X

**DOI:** 10.3390/biology10060450

**Published:** 2021-05-21

**Authors:** Paola Pontecorvi, Francesca Megiorni, Simona Camero, Simona Ceccarelli, Laura Bernardini, Anna Capalbo, Eleni Anastasiadou, Giulia Gerini, Elena Messina, Giorgia Perniola, Pierluigi Benedetti Panici, Paola Grammatico, Antonio Pizzuti, Cinzia Marchese

**Affiliations:** 1 Department of Experimental Medicine, Sapienza University of Rome—Viale Regina Elena 324, 00161 Rome, Italy; paola.pontecorvi@uniroma1.it (P.P.); francesca.megiorni@uniroma1.it (F.M.); simona.ceccarelli@uniroma1.it (S.C.); eleni.anastasiadou@uniroma1.it (E.A.); giulia.gerini@uniroma1.it (G.G.); elena.messina@uniroma1.it (E.M.); antonio.pizzuti@uniroma1.it (A.P.); 2 Department of Maternal and Child Health and Urological Sciences, Sapienza University of Rome—Viale Regina Elena 324, 00161 Rome, Italy; simona.camero@uniroma1.it (S.C.); giorgia.perniola@uniroma1.it (G.P.); pierluigi.benedettipanici@uniroma1.it (P.B.P.); 3 Division of Medical Genetics, IRCCS Casa Sollievo della Sofferenza Foundation-Viale Cappuccini, 1, 71013 San Giovanni Rotondo (FG), Italy; l.bernardini@css-mendel.it (L.B.); a.capalbo@css-mendel.it (A.C.); 4 Division of Medical Genetics, Department of Molecular Medicine, Sapienza University of Rome-San Camillo-Forlanini Hospital, Circonvallazione Gianicolense, 87, 00152 Rome, Italy; paola.grammatico@uniroma1.it

**Keywords:** Mayer–Rokitansky–Küster–Hauser syndrome, PRKX, vaginal keratinocyte, gene expression, cell migration, EMT, *HOX* genes, tissue morphogenesis, genital tract development

## Abstract

**Simple Summary:**

Women affected by Mayer–Rokitansky–Küster–Hauser (MRKH) syndrome show an underdeveloped or absent vagina and uterus, frequently in association with renal defects. The aetiology of MRKH syndrome is controversial. Only a few cases could be related to genetic anomalies, while the vast majority of them lack a precise molecular cause. We found that protein kinase X (PRKX) levels are upregulated in vaginal keratinocytes from MRKH patients with respect to healthy women. Through in vitro investigations, we highlighted that PRKX ectopic overexpression is able to influence vaginal keratinocytes’ cell shape and motility through the induction of epithelial-to-mesenchymal transition (EMT). Moreover, PRKX ectopic overexpression exerts a broad effect on the expression of *HOX* genes, which encode for essential factors implicated in embryo organogenesis. The present study suggests a possible molecular cause for MRKH syndrome and a role for PRKX in vaginal keratinocyte biology.

**Abstract:**

Mayer–Rokitansky–Küster–Hauser (MRKH) syndrome is a rare and complex disease defined by congenital aplasia of the vagina and uterus in 46,XX women, often associated with kidney and urinary tract anomalies. The aetiopathogenesis of MRKH syndrome is still largely unknown. Herein, we investigated the role of selected candidate genes in the aetiopathogenesis of MRKH syndrome, with a focus on *PRKX*, which encodes for protein kinase X. Through RT-qPCR analyses performed on vaginal dimple samples from patients, and principal component analysis (PCA), we highlighted a phenotype-related expression pattern of *PRKX*, *MUC1*, *HOXC8* and *GREB1L* in MRKH patients. By using an in vitro approach, we proved that PRKX ectopic overexpression in a cell model of vaginal keratinocytes promotes cell motility through epithelial-to-mesenchymal transition (EMT) activation, a fundamental process in urogenital tract morphogenesis. Moreover, our findings showed that PRKX upregulation in vaginal keratinocytes is able to affect transcriptional levels of *HOX* genes, implicated in urinary and genital tract development. Our study identified the dysregulation of PRKX expression as a possible molecular cause for MRKH syndrome. Moreover, we propose the specific role of PRKX in vaginal keratinocyte biology as one of the possible mechanisms underlying this complex disease.

## 1. Introduction

Genital tract development involves a plethora of finely regulated processes, in which quantitatively and timely regulated gene expression is crucial. Müllerian ducts (paramesonephric ducts—MD) and Wolffian ducts (mesonephric ducts—WD) are paired ducts running beside the embryonic kidneys. The former develops into the fallopian tubes, uterus, and upper part of the vagina in females, while the latter gives rise to the epididymis, vas deferens, and seminal vesicle in males [1,2]. By the sixth week of gestation, lack of both testis-derived androgens and anti-Müllerian hormone (AMH) in female embryos allows regression of the WD and differentiation of the MD into the female reproductive tract [1]. In the event that MD fail to develop properly, MD anomalies occur, resulting in either a total absence or malformation of the female genital structures.

Mayer–Rokitansky–Küster–Hauser (MRKH) syndrome (OMIM #277000) is a rare and complex disease defined by congenital aplasia of the vagina and uterus in 46,XX women with regular development of secondary sexual features. The syndrome is divided in two categories on the basis of phenotypic associations: MRKH type I, in which patients show isolated malformations of the vagina, cervix and uterus, and the most frequent MRKH type II, in which renal, skeletal, cardiac and/or hearing defects are present along with genital anomalies [3,4]. MRKH syndrome renders it impossible to have normal sexual intercourse, hence infertility and psychological distress for the affected women [5]. Different possible aetiologies for MRKH syndrome should be considered. This condition seems to be justified by a genetic cause only in a small number of patients, with autosomal dominant inheritance and incomplete penetrance, whilst the majority of cases are sporadic [6,7]. The variable expression of phenotypes among MRKH patients and the reports of discordant twin pairs [8,9,10,11] suggests either polygenic/multifactorial or non-genetic aetiologies, such as environmental factors (e.g., xenoestrogens) [12,13] and/or epigenetic alterations.

Concerning epigenetic modifications, Rall et al. demonstrated that DNA methylation might play a pivotal role in MRKH syndrome aetiology by modulating the expression of genes involved in proper patterning of the embryo and partitioning of the female genital tract, such as *HOXA5* and *HOXA9* [14]. Furthermore, Nodale et al. [15] demonstrated deregulated levels of development-related genes in MRKH patients in comparison to healthy women, by observing a significant downregulation of *HOXB2* and *HOXB5* in most MRKH patients and a strong overexpression of *HOXC8* in MRKH type I patients. Moreover, *MUC1* gene, encoding for a heavily glycosylated transmembrane protein, was overexpressed in all MRKH patients screened by microarray compared to healthy controls (15). Recently, Hentrich et al. [16] demonstrated the altered expression of *WNT4*, *WNT5A*, *WNT7A* and *WNT9B* as well as various *HOX* genes in the endometrium of MRKH patients with respect to healthy controls. Moreover, developmental regulators such as *TGFB1* and *EZH2* emerged as central from the analyses. *TGFB1*, encoding for a crucial factor for epithelial-to-mesenchymal transition (EMT) [17], was significantly downregulated in MRKH patients. EMT is necessary for the development and normal functioning of female reproductive organs such as ovaries and uterus [18].

In our latest work, network biology analysis applied to DNA copy number profiling on MRKH patients highlighted the centrality of protein kinase X (PRKX) in the disease [19]. The human *PRKX* gene encodes for a cAMP-dependent serine/threonine kinase, shown to be phylogenetically different from the classical *PKA*, *PKB/AKT*, *PKC*, *SGK* and *PKG* gene families [20]. In recent years, several studies have demonstrated that PRKX is involved in processes such as kidney development and disease, blood maturation, and neural development [21]. The formation of the urinary and reproductive systems is strongly interconnected [22] and many common genes have been implicated in their morphogenesis [23,24]; therefore, the role of PRKX in genital structure development is a fair assumption. However, molecular mechanisms beneath PRKX-related biological function remain unclear.

Considering that only a fraction of MRKH cases can be explained by genetic defects, the contribution of gene expression dysregulation in the aetiology of the syndrome should be further investigated. Herein, we first examined mRNA levels of selected MRKH candidate genes on vaginal dimple samples from patients to highlight a disease-related expression pattern. Then, we focused on the effects of PRKX altered expression on vaginal keratinocyte biology, observing that PRKX might influence EMT and cell migration, and also regulate *HOX* gene expression, which are key events in organogenesis.

## 2. Materials and Methods

### 2.1. Patients

The 34 unrelated patients with MRKH syndrome herein analysed were previously enrolled in a multi-centric study involving Sapienza University and CSS-Mendel Institute of Rome, Italy [19]. Patients’ age at diagnosis ranged between 17 and 49 years old. Diagnosis of MRKH syndrome was determined through standard clinical procedures, as described by Nodale et al. [25]. Patients were classified as having type I or type II MRKH syndrome according to the absence or presence of other genitourinary malformations, kidney anomalies, skeletal malformations, and/or hearing defects.

### 2.2. Cell Cultures and Treatments

Primary cultures of human vaginal keratinocytes were established from 1 cm^2^ full-thickness mucosal biopsy of the vaginal vestibule of 34 MRKH patients (before vaginoplasty) and matching with the vaginal tissue of 4 healthy control women, as previously reported [26,27]. HEK293T and HeLa cell lines (ATCC, Manassas, VA, USA) were both grown in Dulbecco’s modified Eagle’s medium (DMEM; Sigma-Aldrich, St. Louis, MO, USA), supplemented with 10% foetal bovine serum (FBS; Sigma-Aldrich, St. Louis, MO, USA) and 1% penicillin/streptomycin (Aurogene, Rome, Italy). VK2 E6/E7 cell line (ATCC) was established from the normal vaginal mucosal tissue taken from a premenopausal woman undergoing anterior–posterior vaginal repair surgery. VK2 E6/E7 cells were maintained in keratinocyte serum-free medium (K-SFM; GIBCO, Carlsbad, CA, USA) with 0.1 ng/mL human recombinant EGF, 0.05 mg/mL bovine pituitary extract, additional calcium chloride 44.1 mg/L (final concentration 0.4 mM), and 1% penicillin/streptomycin (Aurogene, Rome, Italy). Cells were treated with 100 µM 8-Bromoadenosine 3′,5′-cyclic monophosphate (8-Br-cAMP) (Sigma-Aldrich, St. Louis, MO, USA) in dH_2_O for 1 h.

### 2.3. RNA Extraction, Reverse Transcription and Quantitative Real-Time PCR (RT-qPCR)

Total RNA from primary vaginal cell cultures and cell lines was extracted by using TRIzol reagent (Invitrogen, Carlsbad, CA, USA) and quantified through a NanoDropTM 2000c spectrophotometer (Thermo Fisher Scientific, Waltham, MA, USA). Then, 1–2 µg of RNA was reverse transcribed with the High-Capacity RNA to cDNA Kit (Applied Biosystems by Thermo Fisher Scientific, Waltham, MA, USA), according to the manufacturers’ instructions. Specific gene expression was evaluated by RT-qPCR experiments with TaqMan Gene Expression Assays and Array 96-well Plates (*PRKX*, Hs_00746337_s1; *HOXC8*, Hs00224073_m1; *MUC1*, Hs00159357_m1; *GREB1L*, Hs00227824_m1; *SHOX,* Hs00757860_g1; TaqMan Array Human Hox Genes—by Applied Biosystems). Cyclophilin-A mRNA was used as an endogenous control (*PPIA*, Hs_04194521_s1 by Applied Biosystems). In gene expression studies on MRKH patients, specimens from 4 healthy women (age range: 25–54 years old) were pooled after the accurate evaluation of individual variations of gene expression and used as reference. Samples were run on an AB Step One Plus Real-time PCR System (Applied Biosystems) and results analysed as previously described [28].

### 2.4. Principal Component Analysis (PCA)

PCA is a mathematical algorithm that reduces the dimensionality of the data while retaining most of the variation in the data set. By using a few components, each sample can be represented by relatively few numbers instead of by values for a high number of variables. Samples can then be plotted, making it possible to visually assess similarities and differences between samples and determine whether samples can be grouped [29]. This multivariate analysis approach was applied to gene expression data for *PRKX*, *MUC1*, *HOXC8* and *GREB1L* on 34 MRKH patients. PCA was conducted in Microsoft Excel 2016 using the XLSTAT package (Addinsoft, New York, NY, USA).

### 2.5. PRKX Cloning and Transfection

The *PRKX* coding region was cloned, starting from the pDONR223-PRKX (AddGene, Watertown, MA, USA) plasmid containing the sequence of interest. To amplify the PRKX insert, forward (fw) and reverse (rev) primers were designed to encompass PRKX Open Reading Frame (ORF) (PRKX fw: 5′-GCAAGCTTATGGAGGCGCCCGGGCTGG-3′; PRKX rev: 5′-CCGGAATTCTCAGAAATTCTTGAAGATTTCTAAATC-3′). We included the primers HindIII and EcoRI restriction sites in order to clone the amplified insert in the expression vector pcDNA3.1(+) (Invitrogen, Carlsbad, CA, USA), as well as a stop codon in primer rev. The melting temperature was 60 °C for both primers. The PRKX insert was amplified by qPCR performed by using CloneAmp HiFi PCR Premix 2X (Takara Bio INC, Shiga, Japan) with the following protocol: 95 °C 3′/(98 °C 10″; 60 °C 15″; 72 °C 20″)_×35_/72 °C 10′/4 °C. qPCR product was purified with Wizard SV Gel and a PCR cleaning system (Promega, Madison, WI, USA) and analysed for identity and integrity by electrophoresis run on 1% agarose gel. Amplified PRKX insert and pcDNA3.1(+) vector were both digested with HindIII and EcoRI (New England Biolabs, Ipswich, MA, USA) for 2 h at 37 °C in order to obtain compatible sticky ends. Digestions were then purified with Wizard SV Gel and the PCR cleaning system (Promega, Madison, WI, USA), and dephosphorylation was performed by incubating the digestions’ products with alkaline phosphatase (Roche, Penzberg, Germany) for 30′ at 37 °C. pcDNA3.1(+) vector and PRKX insert were then ligated by a 16 °C o/n incubation with Quick-Stick Ligase (BIOLINE, London, UK). In order to confirm the insertion of PRKX ORF in pcDNA3.1(+) expression vector, ligation product was run on 1% agarose gel which highlighted the presence of a 6505 bp band (5428 bp vector + 1077 bp insert).

We then proceeded with the pcDNA3.1(+)-PRKX transformation of Mix&Go Competent *E. coli* cells (Zymo Research, Irvine, CA, USA), which then were seeded onto LB agar-coated Petri dishes with 100 µg/mL Ampicilline (Sigma-Aldrich, St. Louis, MO, USA) for selection. Picked colonies were expanded and selected for DNA extraction with PureYeld Plasmid Miniprep system (Promega, Madison, WI, USA). Plasmid was then sequenced to exclude the presence of deleterious mutations. Finally, pcDNA3.1(+)-PRKX plasmid midipreps were obtained with GenElute HP Plasmid Midiprep Kit (Sigma, St. Louis, MO, USA).

In order to overexpress PRKX, VK2 E6/E7 cells were transiently transfected with pcDNA3.1(+)-PRKX plasmid using 3 µL of DreamFect Gold (OZbiosciences, Marseille, France) for each microgram of plasmidic DNA, following the manufacturer’s instructions.

### 2.6. Immunofluorescence

VK2 E6/E7 cells were plated in 24-well plates with 2% gelatine-coated glasses (6 × 10^4^ cells/well). After 24 h, cells were transfected with control vector pcDNA3.1(+) or pcDNA3.1(+)-PRKX, and at 48 h after transfection, cells were treated with 8-Br-cAMP for 30′, 1 h, and 18 h. Immunofluorescence of cells fixed in 4% paraformaldehyde was performed as previously described by Ceccarelli et al. [30]. Cells were incubated with PRKX primary antibody (Abcam, Cambridge, UK) for 1 h, followed by specific Texas Red-conjugated secondary antibody (Jackson ImmunoResearch, West Grove, PA, USA) for 30′. Cell nuclei were stained with 1 μg/mL 4′, 6-diamido-2phenylindole dihydrochloride (DAPI, Sigma-Aldrich, St. Louis, MO, USA). Images were acquired with a fluorescence microscope (Olympus BX53, Center Valley, PA, USA) using a 40× magnification.

### 2.7. Protein Extracts and Western Blot Analysis

VK2 E6/E7 transfected with control vector pcDNA3.1(+) or pcDNA3.1(+)-PRKX and treated with 8-Br-cAMP were lysed in RIPA buffer. Total protein extracts (50 μg) were separated on 10% or 15% SDS-PAGE and underwent Western blot analysis, as previously described [31,32]. Membranes were incubated overnight at 4 °C or 1 h at room temperature (RT) with the following primary antibodies: anti-PRKX (Abcam, Cambridge, UK), anti-TWIST (Abcam, Cambridge, UK), anti-SLUG (Santa Cruz Biotechnology, Dallas, TX, USA), anti-E-cadherin (Santa Cruz Biotechnology), anti-β-catenin (Cell Signalling Technology, Danvers, MA, USA) and anti-β-Actin (Santa Cruz Biotechnology, Dallas, TX, USA) as a loading control. Images were acquired by ChemiDoc XRS+ (Bio-Rad, Hercules, CA, USA). Densitometry was performed with ImageJ software [33].

### 2.8. Cell Migration Assay

Transwell cell migration assays were performed using BD Falcon Cell Culture Inserts. A total of 2.7 × 10^5^ VK2 E6/E7 cells per well were seeded onto 6-well plates in K-SFM without antibiotics and transfected with pcDNA3.1(+) or pcDNA3.1(+)-PRKX. At 48 h post-transfection, cells were treated with 8-Br-cAMP for 1 h and then trypsinised. Then, 5 × 10^4^ cells were placed in the insert and allowed to migrate for 24 h. The outer chamber was filled with 500 μL of DMEM-F12 (Sigma-Aldrich, St. Louis, MO, USA) containing 5% FBS. After incubation, cells were fixed with methanol and stained with crystal violet [34]. Non-migrating cells on the upper surface of the insert were accurately removed with cotton swabs. The 8 µm pore membranes were cut from the inserts and mounted on microscopy glasses. Pictures of 10× magnification of four different fields for treatment point were taken with EVOS XL Core Imaging System (Thermo Fisher Scientific, Waltham, MA, USA) and the total number of cells were determined using ImageJ software [33].

### 2.9. Protein–Protein Interaction (PPI) Network Analysis

System biology analysis was conducted using the STRING 9.1 database (version 11.0; http://string-db.org/ (accessed on 10 May 2021) [35,36] to combine both known and predicted protein–protein interactions. Proteins encoded by the *HOX* genes that were significantly deregulated by PRKX overexpression in the TaqMan Array Plate assay, PRKX and its substrates CREB1, SMAD6 and PKD1, were used as inputs. The interactions were obtained with a confidence score of 0.150. The network created by STRING 9.1 was analysed by employing the Cytoscape 3.8.0 platform [37], using the additional software plugins CentiScaPe 2.2 [38] and yFiles Layout Algorithms (yWorks GmbH, Tübingen, Germany), as previously described [19].

### 2.10. Statistics

For each experiment, at least two replicates were performed, and all results are expressed as the mean ± SD. Statistical significance was calculated with GraphPad Prism 8.0.1 (GraphPad Software Inc., San Diego, CA, USA) using Student’s *t*-test. *p*-values less than 0.05 were considered statistically significant.

## 3. Results

### 3.1. Investigating PRKX and Selected Candidate Genes Expression in MRKH Patients

**Figure 1:**
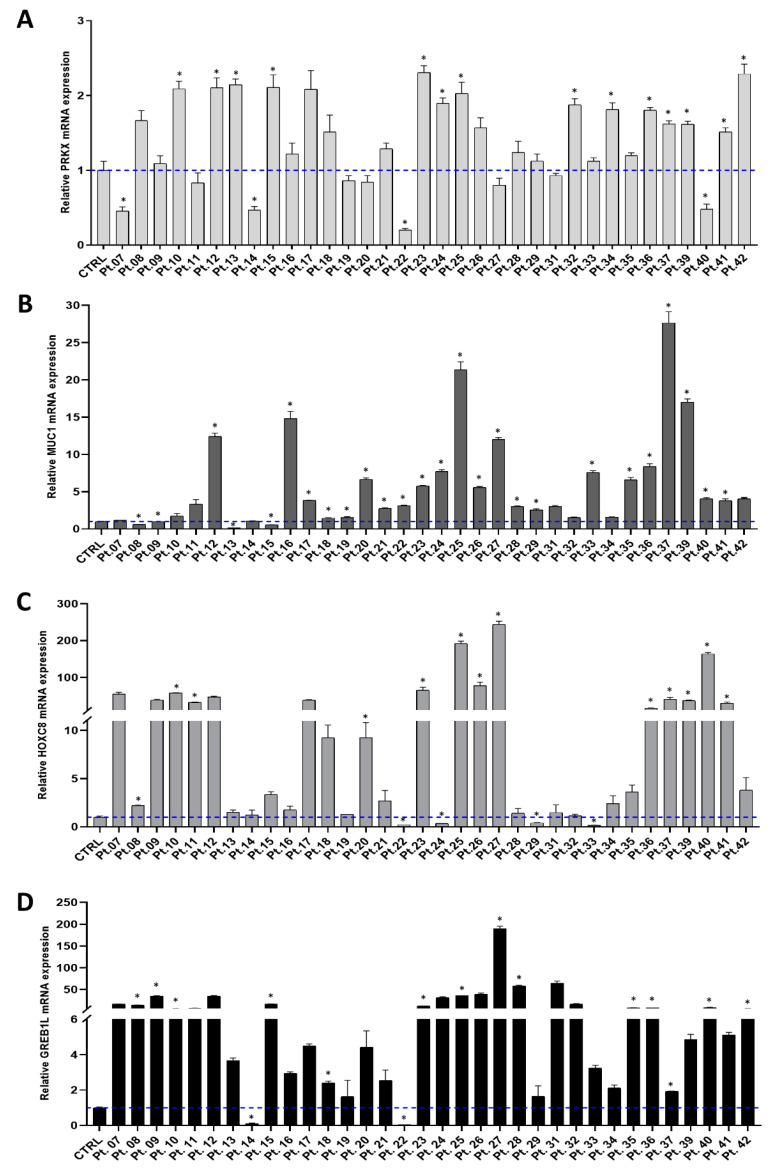
Candidate gene expressions in the cohort of MRKH patients. RT-qPCR analysis performed on RNA extracted from primary vaginal keratinocytes showing PRKX (**A**), MUC1 (**B**), HOXC8 (**C**) and GREB1L (**D**) relative mRNA levels with respect to a pool of 4 healthy control women, indicated with a blue dashed line (mean ± SD; *n* = 3; * *p* < 0.05; Student’s *t*-test).

In order to confirm altered *PRKX* gene expression in MRKH syndrome, as suggested by our recently published work [19], we extended PRKX mRNA expression analysis to the entire cohort, investigating 34 MRKH patients and a pool of 4 healthy control women. The difficulty of obtaining vaginal biopsies from healthy young women, both for the physical and psychological distress linked to the procedure, accounted for the low number of controls. To examine PRKX mRNA levels, we performed RT-qPCR experiments on RNA extracted from vaginal vestibule keratinocytes by using specific TaqMan PRKX primers/probe. As illustrated in Figure 1A, *PRKX* expression showed a moderate but significant increase in 14 out of 34 MRKH patients compared to the pooled control (ranging from a 1.5- to 2.3-fold increase; *p* < 0.05). Only in four patients was *PRKX* expression significantly decreased compared to the control group (*p* < 0.05). We then focused our attention on the expression of other genes selected from the literature which are likely to play a role in MRKH syndrome phenotype determination: *MUC1*, *HOXC8* and *GREB1L* [15,39].

Regarding *MUC1* mRNA levels, they were significantly deregulated in 26 out of 34 MRKH patients compared to healthy controls, showing a trend of *MUC1* overexpression with a maximum of 28-fold through the entire cohort (*p* < 0.05; Figure 1B).

Concerning *HOXC8*, we highlighted a significant deregulation at mRNA level in 50% of patients versus controls (*p* < 0.05). Notably, the fold change ranged between 0.17 and 243, with five MRKH women in which HOXC8 expression was nearly undetectable, making it the most variable gene among those analysed (Figure 1C).

Looking at *GREB1L* expression, we found it significantly deregulated in 47% of MRKH patients compared to controls, ranging from a 0.05- to 189-fold change (*p* < 0.05; Figure 1D).

Patients 32, 33, 34, 35, 36, 37, 39, 40 and 41 were previously screened for chromosomal aberrations with the array-CGH and MLPA test [19]. Patient 36 showed a deletion in 7q12 chromosomal region, and patient 39 presented a chromosomal deletion in 16p11.2, but these regions do not include any of the analysed candidate genes.

The high expression variability of selected genes in each MRKH patient and the high number of patients posed a challenge in the establishment of a correlation between gene expression pattern and MRKH patients’ phenotypic features. To overcome this difficulty, we applied a statistical approach to study the RT-qPCR data by using PCA analysis.

### 3.2. Principal Component Analysis (PCA) Suggests Correlations between Expression Pattern of Selected Candidate Genes and MRKH Phenotypes

**Figure 2:**
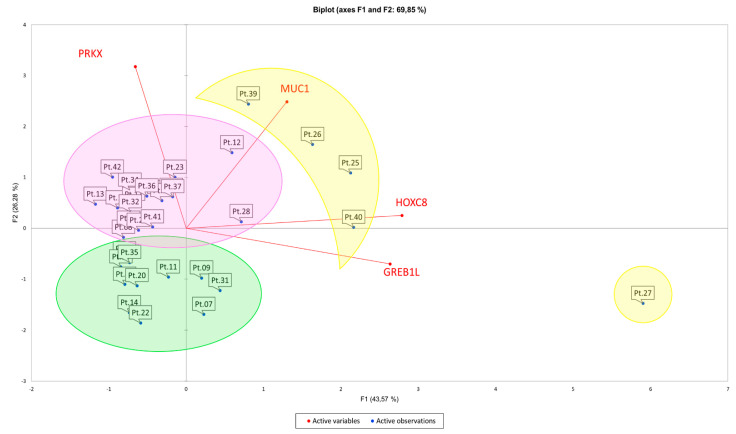
PCA on MRKH patients’ gene expression data. Graphical illustration of the 3 clusters retrieved from PCA. Cluster 1 (purple circle) contains 66.6% of MRKH type I, cluster 2 (green circle) includes 63.6% of MRKH type II, and cluster 3 (yellow circle) encompasses 5 outliers.

In order to establish a correlation between MRKH syndrome phenotype and gene expression pattern, we performed PCA on data from RT-qPCRs for *PRKX*, *MUC1*, *HOXC8* and *GREB1L* loci. The aim of this analysis was to identify gene expression profiles recurring among MRKH patients with similar phenotype features. Indeed, the discovery of a commonly altered gene expression pattern could help in better understanding the molecular basis of a high number of MRKH cases, especially when genetic aberrations were not detected. All the statistical calculations are listed in Appendix A. This analysis allowed us to retrieve three different clusters, two of which contained MRKH patients with shared syndrome types (Table 1 and Figure 2).

In particular, cluster 1 (purple circle) consisted of 18 patients, and 12 of them were MRKH type I (66.6%); cluster 2 (green circle) included 11 patients and 7 of them were MRKH type II (63.6%). Cluster 3 (yellow circle) encompassed 5 outliers, for which we could not find any significant correlation between expression pattern and disease phenotype. Our findings suggest a correlation between the concurrent variation of *PRKX*, *MUC1*, *HOXC8* and *GREB1L* mRNA levels and MRKH clinical features. Moreover, *HOXC8* and *GREB1L* variations seem to be positively related with each other. However, this is only a statistical prediction, and, as the Kaiser–Meyer–Olkin test suggested, a higher number of patients and genes should be included in the analysis to confirm the results [40].

### 3.3. Assessment of Basal PRKX Expression Levels in Selected Human Cell Lines and PRKX Expression Vector’s Functionality

**Figure 3:**
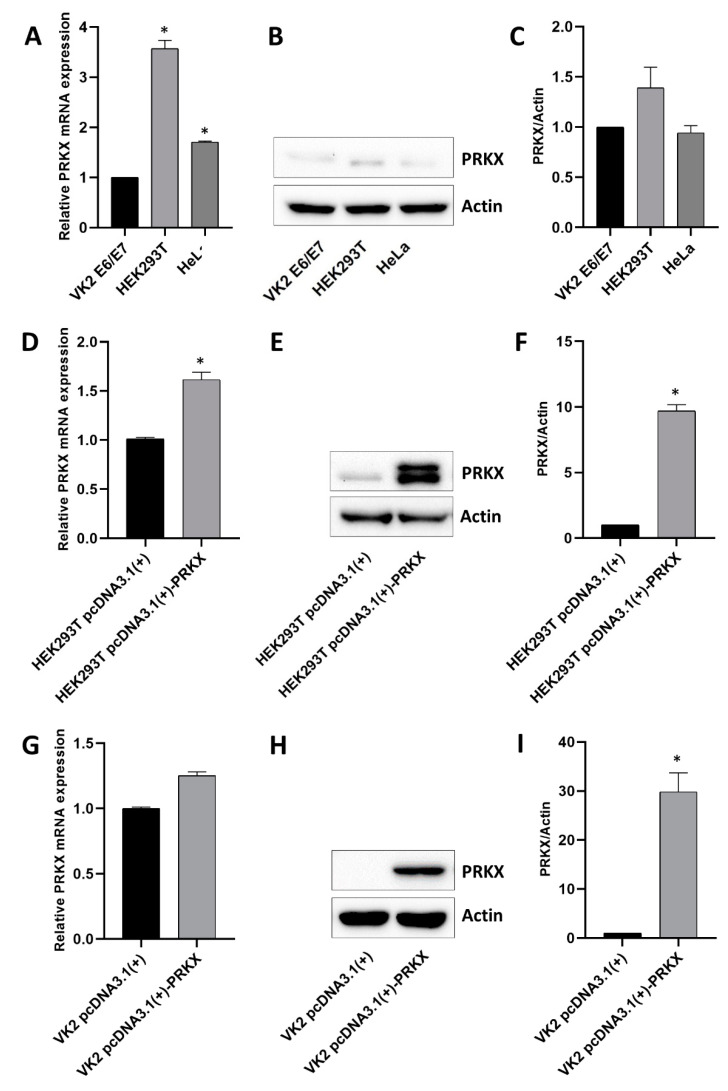
PRKX basal expression in selected human cell lines and PRKX expression vector assessment. (**A**) RT-qPCR analysis performed on RNA extracted from VK2 E6/E7, HEK293T and HeLa cell lines showing PRKX mRNA levels relative fold change. (**B**) Representative Western blot for PRKX in VK2 E6/E7, HEK293T and HeLa cell lines (β-Actin was used as loading control) and densitometric analysis (**C**) (mean ± SD; *n* = 2; * *p* < 0.05; Student’s *t*-test). (**D**) RT-qPCR analysis showing PRKX mRNA levels in HEK293T transfected with PRKX expression vectors (pcDNA3.1(+)-PRKX) or empty vectors as controls (pcDNA3.1(+)). (**E**) Representative Western blot for PRKX in HEK293T transfected with pcDNA3.1(+)-PRKX or pcDNA3.1(+) as control (β-Actin was used as loading control) and densitometric analysis (**F**) (mean ± SD; *n* = 2; * *p* < 0.05; Student’s *t*-test). (**G**) RT-qPCR analysis showing PRKX mRNA levels in VK2 E6/E7 transfected with PRKX expression vector (pcDNA3.1(+)-PRKX) or empty vector as control (pcDNA3.1(+)). (**H**) Representative Western blot for PRKX in VK2 E6/E7 transfected with pcDNA3.1(+)-PRKX or pcDNA3.1(+) as control (β-Actin was used as loading control) and densitometric analysis (**I**) (mean ± SD; *n* = 2; * *p* < 0.05; Student’s *t*-test). The original Western Blot images can be found in Figure S1.

Given the centrality of PRKX in our previous work [19], we decided to deepen our insight into its molecular role by using in vitro models. First, we considered three representative cell lines suitable for MRKH disease model: human vaginal keratinocytes (VK2 E6/E7), human embryonic kidney cells (HEK293T) and human cervical cancer cells (HeLa). We tested these cell lines for *PRKX* expression at the mRNA and protein level (Figure 3A–C).

Among the three, we selected VK2 E6/E7 cells for further studies, even though they showed the lowest levels of PRKX. In fact, they were the same type of cell isolated from MRKH patients’ biopsies and displayed features of primary immortalised cells, not cancer-derived cells.

We found altered PRKX mRNA expression throughout our cohort of MRKH patients; therefore, we hypothesised that increased levels of PRKX levels may somehow influence the molecular mechanisms underneath MRKH syndrome determination. The first step in the investigation this supposition was cloning the *PRKX* coding sequence into an expression vector, hereafter indicated as pcDNA3.1(+)-PRKX, in order to overexpress PRKX levels in our cellular models. HEK293T is frequently used in transfection experiments with verified protocols, therefore we chose it to assess the transcriptional and translational functionality of the PRKX plasmid. Transfection efficiency was tested by GFP vector expression analysis and was about 80% (data not shown). As showed in Figure 3D, the PRKX mRNA fold increase was around 1.6 for pcDNA3.1(+)-PRKX transfected cells compared to control (*p* < 0.05), whilst protein levels rose about 10-fold after pcDNA3.1(+)-PRKX plasmid transfection (*p* < 0.05; Figure 3E,F), confirming the effectiveness of the plasmid.

Considering that the role of PRKX in kidney development and diseases has already been investigated by other groups using HEK293T and other renal models [20,21], we focused our attention on vaginal keratinocytes cell, despite the limitations related to their nature of differentiated adult cells.

We performed PRKX overexpression experiments on VK2E6/E7 cells, which have proven to be quite reluctant to transfection. After protocol optimisation, transfection efficiency was established at 45% by GFP vector expression assay (data not shown). Figure 3G shows that PRKX mRNA levels after plasmid transfection rose to a maximum of 1.3-fold, resulting in about a 30-fold increase in PRKX protein expression (*p* < 0.05; Figure 3H,I). Notably, pcDNA3.1(+)-PRKX transfection in VK2 E6/E7 determined an increase in PRKX mRNA levels comparable to that observed in MRKH patients with respect to healthy controls.

Although the transfection efficiency was suboptimal for VK2 E6/E7, this was confirmed by a lower mRNA fold increase in transfected VK2 E6/E7 than in HEK293T; the relative protein fold increase in VK2 E6/E7 transfected with pcDNA3.1(+)-PRKX was higher than that observed in HEK293T. This could be explained by the lower basal levels of PRKX in VK2 E6/E7 with respect to HEK293T.

Moreover, the weak increase in PRKX mRNA levels both in HEK293T and in VK2 E6/E7 at 48 h after transfection seems not to completely justify the substantial rising of PRKX protein levels. However, it should be noted that, at the same time point, mRNA and protein levels are not always linearly correlated. Indeed, post-transcriptional and post-translational modifications may influence the efficiency of protein production and accumulation [41].

### 3.4. PRKX Overexpression in VK2 E6/E7 Cells Induces Cell Morphology Modifications

**Figure 4:**
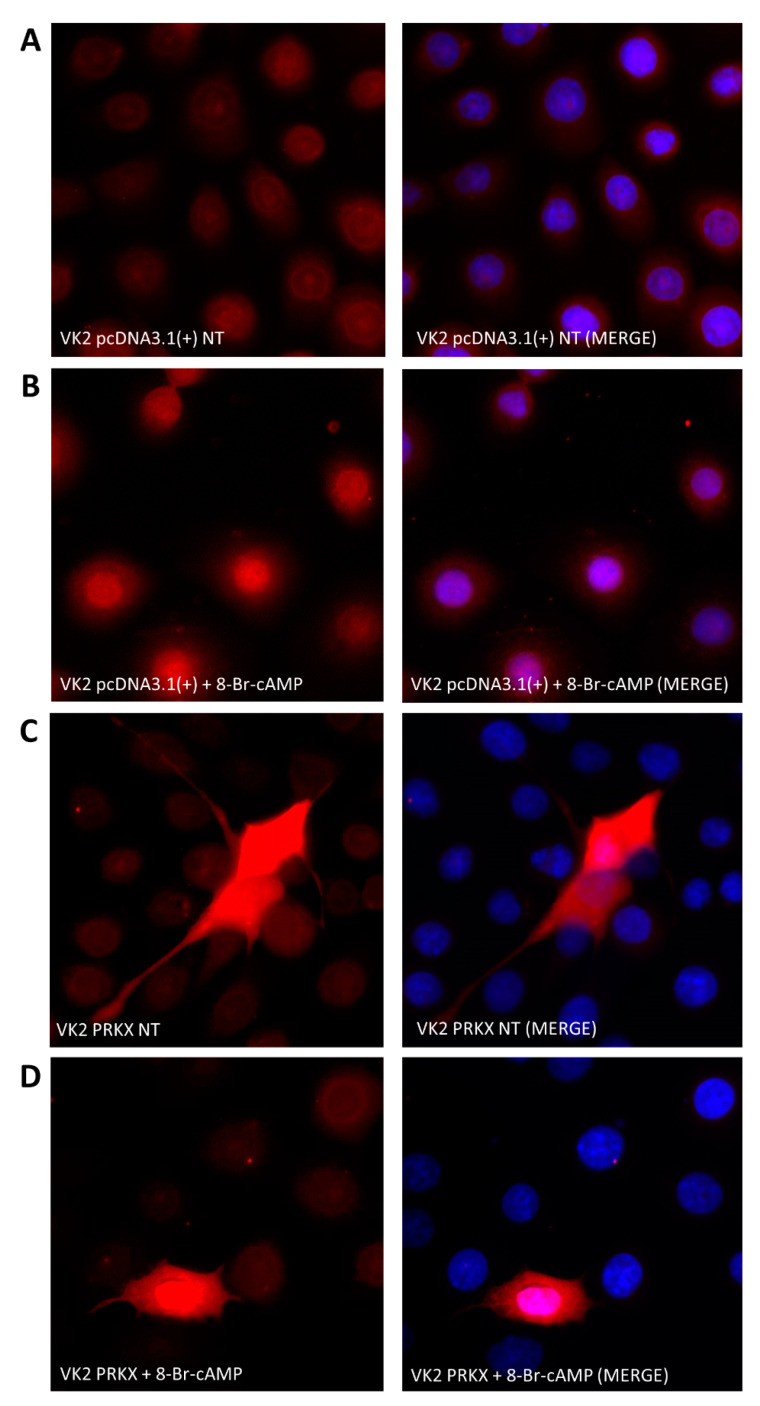
PRKX overexpression and activation in human vaginal keratinocytes. Immunofluorescence showing VK2 E6/E7 transfected with empty vector (pcDNA3.1(+)) in the presence or absence of 8-Br-cAMP (**A**,**B**) and VK2 E6/E7 transfected with PRKX expression vector (pcDNA3.1(+)-PRKX) with or without 8-Br-cAMP treatment (**C**,**D**). PRKX is stained with Texas Red, nuclei are stained in blue with DAPI (20× magnification).

PRKX is a cAMP-dependent Ser/Thr protein kinase activated upon the binding of cAMP to its regulatory subunit which determines its detachment [21]. In order to test the full functionality of plasmid-derived PRKX, after its overexpression in VK2 E6/E7, we treated the cells with 8-Br-cAMP, an analogous of cyclic AMP. We performed a dose–time response curve in order to investigate the 8-Br-cAMP effects on pcDNA3.1(+)-PRKX transfected VK2 E6/E7 through immunofluorescence assays (data not shown). We set the treatment point at 100 µM 8-Br-cAMP for a minimum of 1 h, because we saw the maximum PRKX cytoplasm to nucleus translocation and nuclear signal at that point. Figure 4A,B shows the nuclear translocation of endogenous PRKX upon 1 h 8-Br-cAMP treatment in control VK2 E6/E7.

In Figure 4C,D, a stronger PRKX signal is recognisable in pcDNA3.1(+)-PRKX transfected cells compared to controls, which after 1 h 8-Br-cAMP treatment is redistributed from cytoplasm to the nucleus. Furthermore, the immunofluorescence assay highlighted a change in cell morphology in VK2 E6/E7 transfected with pcDNA3.1(+)-PRKX compared to controls (Figure 4A–D). These cells appeared elongated, with accentuated spindle shape and many cell membrane protrusions, all features of migrating cells.

### 3.5. PRKX Overexpression Promotes VK2 E6/E7 Cells Migration as a Consequence of EMT Induction

**Figure 5:**
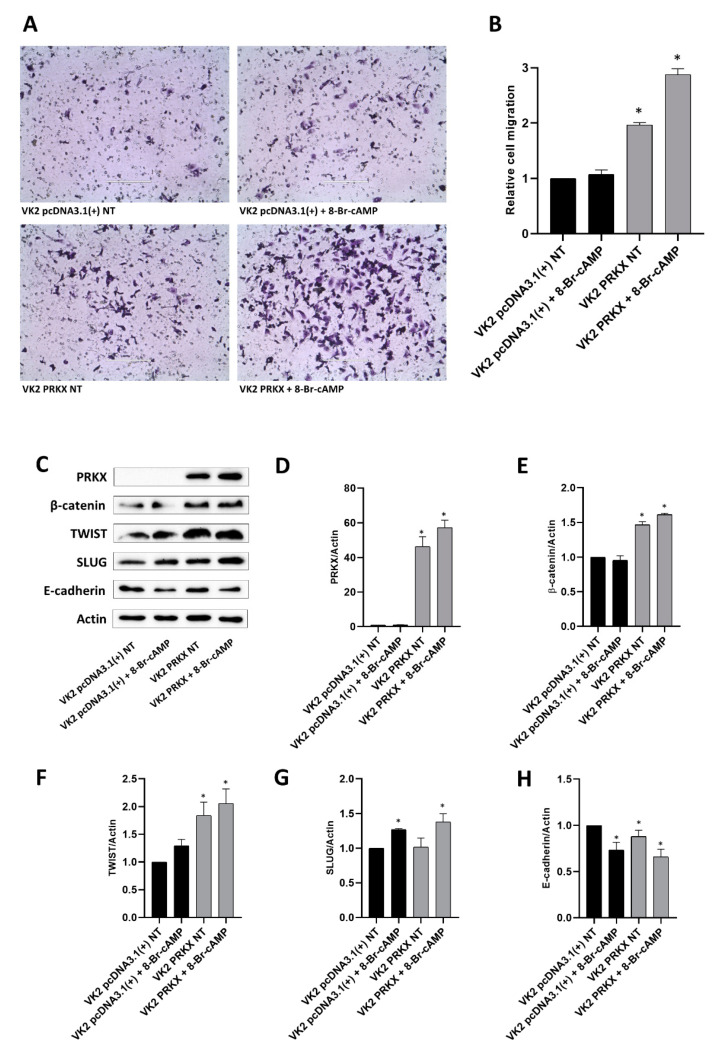
PRKX overexpression promotes the migration and EMT of human vaginal keratinocytes. (**A**) Representative pictures of crystal violet-stained VK2 E6/E7 migrated through 8 µm porous membranes, transfected with pcDNA3.1(+) or pcDNA3.1(+)-PRKX in the presence or absence of 8-Br-cAMP (10× magnification). (**B**) Relative fold increase in cell migration resulted from ImageJ total cell count. (mean ± SD; *n* = 2; * *p* < 0.05; Student’s *t*-test). (**C**) Representative Western blot for PRKX, β-catenin, TWIST, SLUG and E-cadherin in VK2 E6/E7 transfected with pcDNA3.1(+)-PRKX or pcDNA3.1(+) as control in presence or absence of 8-Br-cAMP (β-Actin was used as loading control). (**D**–**H**) Densitometric analysis (mean ± SD; *n* = 2; * *p* < 0.05; Student’s *t*-test). The original Western blot images can be found in Figure S1.

To demonstrate that PRKX overexpression may promote cell motility, we performed a transwell migration assay for the same points analysed through immunofluorescence. After 48 h pcDNA3.1(+)-PRKX transfection and 1 h 8-Br-cAMP treatment, we loaded VK2 E6/E7 cell suspension in the inserts and let them migrate overnight. Figure 5A shows that the overexpression of PRKX alone significantly induced the migration of VK2 E6/E7 cells and that 8-Br-cAMP treatment reinforced this effect. The results were confirmed by the relative cell count presented in Figure 5B, underlining a twofold (*p* < 0.05) and a threefold increase (*p* < 0.05) in cell migration for VK2 E6/E7 pcDNA3.1(+)-PRKX transfected in the absence or presence of 8-Br-cAMP, respectively.

In order to investigate the cellular mechanism at the basis of PRKX-induced VK2 E6/E7 cell migration, we performed Western blot analysis for EMT markers. EMT is a crucial cellular mechanism at the basis of migration and organisation of many cell types into tissues and organs during embryogenesis [42]. To prove the implication of EMT in PRKX-mediated cell migration, we transfected VK2 E6/E7 cells with pcDNA3.1(+)-PRKX or empty vector and treated the cells with 8-Br-cAMP for 1 h. Following the same timing we used for the migration assay, we waited overnight before collecting the cells for protein extraction. The Western blot analysis illustrated in Figure 5C and the relative densitometric analyses (Figure 5D–H) show a significant upregulation of β-catenin, TWIST and SLUG in VK2 E6/E7 pcDNA3.1(+)-PRKX transfected cells compared to controls, with a greater effect observed in the presence of 8-Br-cAMP treatment. Furthermore, E-cadherin was modulated by the overexpression of PRKX, and even if this modulation was slight, the densitometric analysis allowed us to appreciate the significance of the result (Figure 5H).

We observed that PRKX overexpression alone promotes EMT, inducing changes in cell morphology and migration. This effect is additional to the one possibly deriving from the cAMP/PKA pathway [43,44], because the maximum results we observed were in VK2 E6/E7 pcDNA3.1(+)-PRKX treated with 8-Br-cAMP.

### 3.6. PRKX Overexpression in VK2 E6/E7 Cells Affects HOX Gene Expression

**Figure 6:**
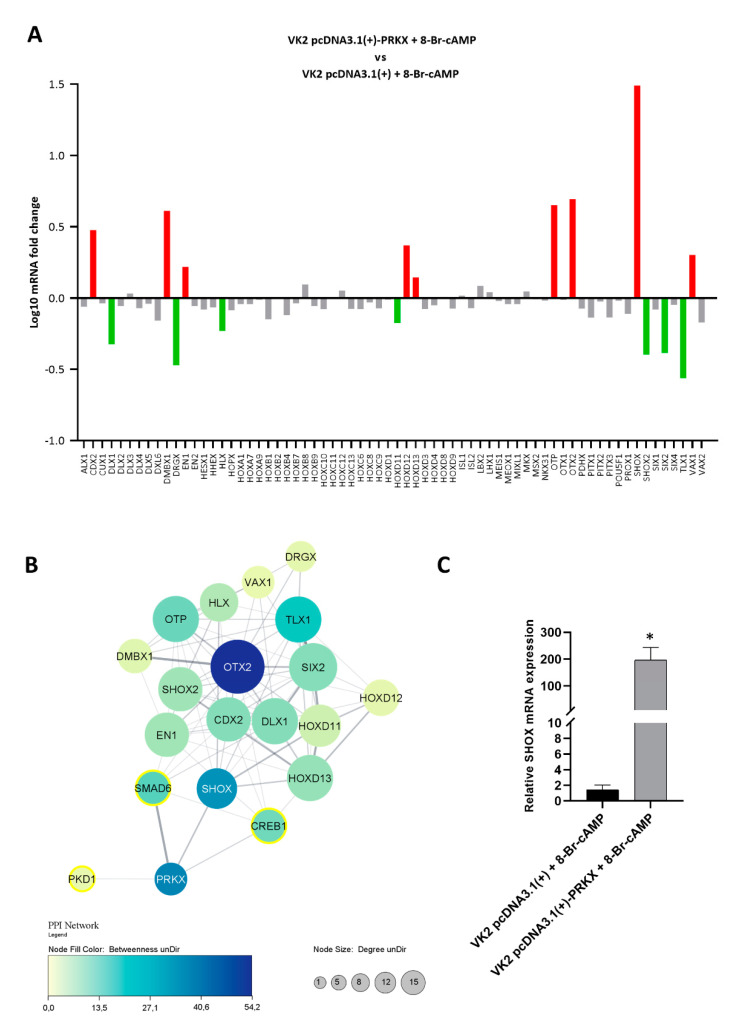
PRKX overexpression influences the expression of HOX genes in VK2 E6/E7 cells. (**A**) Recapitulating graph of RT-qPCR screening for HOX gene mRNA level alterations in VK2 E6/E7 cells transfected with pcDNA3.1(+)-PRKX after 8-Br-cAMP treatment. Relative fold changes represented on a log10 scale are referred to as VK2 E6/E7 cells transfected with empty vector as the baseline. Gene expression variation was considered significant for fold changes ≤0.6 and ≥1.3. Significantly upregulated and downregulated genes are indicated as red and green columns, respectively. (**B**) PPI network showing interactions between PRKX, its principal downstream substrates SMAD6, CREB1, PKD1, and HOX genes’ products that were deregulated from the RT-qPCR array analysis. Node size is directly proportional to connectivity degree and node colour is correlated to betweenness value, with a darker colour corresponding to a higher value. PRKX substrates are circled in yellow. (**C**) RT-qPCR analysis showing SHOX mRNA levels in VK2 E6/E7 transfected with PRKX expression vector (pcDNA3.1(+)-PRKX) or empty vector as control (pcDNA3.1(+)) and treated with 8-Br-cAMP (mean ± SD; *n* = 2; * *p* < 0.05).

As our studies have highlighted, PRKX overexpression may influence vaginal keratinocyte migration through EMT induction. However, we assumed that PRKX molecular action could be broader. We hypothesised that because PRKX translocates to the nucleus upon 8-Br-cAMP treatment, it may directly act on gene expression. *HOX* genes are important regulators of embryogenesis and organogenesis, therefore we screened VK2 E6/E7 cells transfected with pcDNA3.1(+)-PRKX or empty vector after 8-Br-cAMP treatment through an RT-qPCR array for 84 developmental genes. Interestingly, our investigations underlined altered mRNA levels of specific *HOX* genes implicated in urinary and genital tract development (Figure 6A).

We established an arbitrary cut-off for gene expression variation significance (≤0.6- and ≥1.3-fold) and highlighted a substantial upregulation of *CDX2*, *DMBX1*, *EN1*, *HOXD12*, *HOXD13*, *OTP*, *OTX2*, *SHOX* and *VAX1* with fold changes ranging from 1.39 to 30.8 (in red in the graph) in VK2 E6/E7 transfected with pcDNA3.1(+)-PRKX with respect to the control. Moreover, we observed a significant downregulation of *DLX1*, *DRGX*, *HLX*, *HOXD11*, *SHOX2*, *SIX2* and *TLX1* with fold changes ranging between 0.66 and 0.27 (in green in the graph). In order to underline the connection of PRKX and its principal downstream targets *SMAD6*, *CREB1* and *PKD1* [21] with the upregulated and downregulated developmental genes, we built a protein–protein interaction (PPI) network including 20 nodes and 80 edges by applying STRING data to Cytoscape software (Figure 6B). Interestingly, the PPI network unveiled an interaction of PRKX with SHOX. The evidence suggests that a functional link is the co-expression of putative homologs in other organisms (i.e., *Dictyostelium discoideum*). Many links rising from SHOX towards the other deregulated *HOX* genes indicate that a minimal perturbation operated by PRKX overexpression may influence a notable number of downstream genes. Indeed, in this network, SHOX shows high connectivity degree (number of links with other nodes) and betweenness value (capability of a protein to connect distant proteins). Moreover, many interactions were found between *CREB1*, *SMAD6* and several *HOX* genes in the network. The fact that PRKX may act also through SHOX was an intriguing hypothesis, therefore we validated the remarkable upregulation of SHOX after PRKX overexpression in an independent experiment. Through RT-qPCR, we observed an increase in SHOX mRNA levels up to 164-fold in VK2 E6/E7 transfected with pcDNA3.1(+)-PRKX with respect to the control (Figure 6C). Taken together, these results further indicate a role for PRKX in MRKH syndrome determination, which should be confirmed by future in vivo studies.

## 4. Discussion

MRKH syndrome is a complex disease and the specific molecular mechanisms contributing to its determination are still poorly understood. With our latest work, we have contributed to shedding some light on the genetic aetiology of MRKH syndrome [19]. Indeed, in one patient of our MRKH cohort, we highlighted a duplication in the Xp22.33 chromosomal region that contains the *PRKX* gene, encoding for a serine/threonine kinase implicated in renal epithelium morphogenesis [20,45,46,47] and kidney development [48,49]. Notably, through a network medicine analysis [50], we highlighted a possible central role for PRKX in MRKH determination.

In a previous study from our group [15], we highlighted genes that showed deregulated mRNA levels in MRKH patients compared to healthy controls, namely, *MUC1* and *HOXC8*. MUC1 protein seems to interact with β-catenin, an essential mediator of canonical Wnt signalling that plays a critical role in female genital tract development through its direct and indirect action on AMH and its receptor [51,52]. MUC1 overexpression stabilises β-catenin and enhances its nuclear translocation [53]. Moreover, its overexpression during foetal development might induce improper AMH-AMHR2 activation, thus causing a partial MD regression. HOXC8 is known to be involved in correct patterning of the axial skeleton along the antero-posterior axis during early embryogenesis [54] and in kidney differentiation [55]. Concerning *GREB1L*, recently, it has been designated as a gene frequently involved in hereditary urogenital dysplasia and as a significant contributor in MRKH syndrome, more precisely in the MRKH type II subgroup [39,56,57,58,59]. The *GREB1L* gene product is thought to be implicated in retinoic acid receptor (RAR) signalling due to the high similarity with its paralog *GREB1* [56], and dysregulated RAR signalling has previously been associated with kidney development [60,61] and MD development in mice [62]. For the reasons stated above, we investigated *PRKX*, *MUC1*, *HOXC8* and *GREB1L* expression patterns in our cohort of MRKH patients through RT-qPCR. Firstly, we evaluated *PRKX* gene expression levels on vaginal dimple samples from 34 selected MRKH women of our cohort, which underwent vaginoplasty with autologous in vitro cultured vaginal tissue [25,26,27,63,64,65,66]. We highlighted significantly altered *PRKX* mRNA levels in 53% of patients compared to healthy controls, observing an upregulation of *PRKX* in the majority of them.

In agreement with the observations of Nodale et al. [15], we found *MUC1* upregulation in MRKH patients as well as the highest *HOXC8* mRNA levels in MRKH type I patients. In contrast, patients showing significantly lower levels of *HOXC8* than controls (Pt. 22, 24, 29 and 33) are all MRKH type II (*p* < 0.05), suggesting a possible type-specific expression pattern of *HOXC8*. It has been hypothesised that a consistent overexpression of *HOXC8* might be a compensatory mechanism to offset the decreased expression of other developmental genes, which can limit the onset of other malformations, mainly those involving the renal system, and then producing a milder phenotype (MRKH type I) [15]. Regarding *GREB1L*, about 41% of MRKH patients showed a significant upregulation compared to controls, suggesting that a deregulation of GREB1L protein expression may determine an imbalance in RAR signalling, which, in turn, results in genital tract anomalies. The only two patients presenting significant lower levels of *GREB1L* mRNA relatively to healthy controls (*p* < 0.05) were MRKH type II (Patient 14 and 22), with unilateral renal agenesis. This observation seems to confirm the role of *GREB1L* haploinsufficiency in kidney anomalies [39,56].

Through PCA, we analysed RT-qPCR data and found that, based on *PRKX*, *MUC1*, *HOXC8* and *GREB1L* expression patterns, our MRKH cohort was divided in mainly two subgroups. Each group had a prevalence of MRKH type I or MRKH type II, indicating a possible association between candidate gene expression and disease phenotype.

PRKX has never been correlated to female genital tract anomalies before, therefore we decided to deeply explore its molecular and biological function in MRKH syndrome. Specifically, we chose to investigate the effects of the overexpression of *PRKX*, because we found a duplication of this gene in one MRKH patient [19] and observed its general upregulation through our MRKH cohort. We found that PRKX overexpression in a human vaginal keratinocyte model promotes cell migration. This effect has already been observed by Li et al., on renal cells [20], but interestingly we showed the same result in vaginal cells, thus stressing the PRKX concomitant role in urinary and genital tract development. Additionally, we demonstrated that the increased cell motility resulting from PRKX overexpression is mediated by partial EMT. A variety of biomarkers have been used to demonstrate the shift of polarised epithelial cells to an unpolarised migratory mesenchymal state [67]. The most visible protein signatures of EMT include at least one changing cytoskeletal protein, transcription factor, and adherence protein [67]. Indeed, our results showed a slight change in E-cadherin levels with significantly increased expression of EMT key transcription factors, such as β-catenin, TWIST and SLUG, in vaginal keratinocytes transfected with PRKX expression vector and treated with 8-Br-cAMP, a cAMP analogous.

Two considerations must be made: (1) the simultaneous expression of epithelial and mesenchymal markers seemed not to completely justify the entity of cell migration observed herein; and (2) 8-Br-cAMP treatment alone may promote discordant effects, such as a certain degree of EMT, or even the expression of epithelial markers, by acting on PKA [43,44,68]. Many studies have demonstrated that EMT can occur to various extents, therefore it is no more considered as a binary process during which a mature epithelial cell shifts to a fully mesenchymal phenotype, but as a continuum of cell types co-expressing a variety of epithelial and mesenchymal features (partial EMT) [69,70]. This simultaneous adoption of migratory capacity while maintaining intercellular adhesion occurs in numerous developmental systems and is associated with the collective migration of many cell populations [71]. Finally, the cAMP role in EMT promotion is controversial, depending on the cell type, the nature of the EMT inducers, and the maximum levels of the intracellular cAMP obtained [72].

Moreover, we decided to model the direct effects of PRKX on developmental gene expression, specifically *HOX* genes, in a vaginal keratinocyte cell line using an RT-qPCR array. PRKX ectopic overexpression was able to upregulate mRNA levels of *CDX2*, a gene involved in urogenital sinus formation and RAR signal regulation, which plays a role in MD development and differentiation [73]. Similarly, PRKX seemed to determine the upregulation of *DMBX1*, which is expressed in the urogenital system and testis [74], as well as the positive modulation of *VAX1* in VK2 E6/E7 cells. *VAX1* interacts with EMX2 protein [75], which was found to be indispensable for the formation of both MD and WD in mice [76]. Moreover, in our study, ectopic PRKX overexpression seemed to upregulate *HOXD13*, whose alteration has been linked to syndromes affecting genitourinary development [77], renal agenesis [78], and limb malformations such as synpolydactyly and polydactyly [79]. PRKX overexpression is also able to influence the mRNA levels of *HOXD11* and *HOXD12*, which regulate branching morphogenesis of the ureteric bud in the developing kidney [80,81]. The most interesting finding of the reported RT-qPCR array analysis is that pcDNA3.1(+)-PRKX transfection determines a significant upregulation of *SHOX* in VK2 E6/E7 cells. The *SHOX* gene plays a role in bone development and growth and is closely involved in the morphogenesis of the urogenital system [82]. *SHOX* duplications have been found in five MRKH type I patients [82], therefore our results might suggest a possible hidden connection between *PRKX* and *SHOX* expression in MRKH phenotype determination. Regarding PRKX-downregulated *HOX* genes, *DLX1*, which interacts with β-catenin and activates β-catenin/TCF signalling [83], *SHOX2*, involved in heart, skeleton and limbs development [84], *SIX2*, a transcription factor required for the maintenance of metanephric tubule progenitors [85], and *TLX1*, a critical regulator of RA metabolism [86], were negatively modulated in VK2 E6/E7 PRKX-transfected cells. Concerning *DRGX, EN1*, *HLX*, *OTP*, *OTX2* genes, whose mRNA levels demonstrated a likely significant variation upon PRKX ectopic induction, it is not yet evident how to link their function to MRKH-related pathways or developmental processes.

PRKX similarity with PKA suggests that CREB family transcription factors may be among its nuclear targets [21]. Indeed, PRKX was shown to be capable of activating CREB-dependent transcription in vitro [87]. However, in our array plate, the expression of genes, which were predicted to have CRE responsive elements in their promoter (*DXL6*, *HOXC10*, *ISL2*, *LHX1*, *MSX2*) [88], were not directly modulated by PRKX overexpression. Two reasons may be considered: these *HOX* genes are epigenetically silenced in VK2 E6/E7 cells, or the mechanism of action of PRKX does not implicate CREB phosphorylation in this cellular model. Nevertheless, PRKX substates, such as CREB1 and SMAD6, showed many links with *HOX* genes in the PPI network. The majority of the interactions are based on the presence of putative homologs interacting in other organisms, but the relationship of CREB1 with *HOXD13* has been experimentally validated. This suggest that PRKX may indirectly influence *HOX* gene expression through the phosphorylation of its targets. The most interesting finding that we highlighted through PPI network analysis is that, among the dysregulated genes, PRKX is predicted to interact directly with *SHOX*, which in turn is highly connected with the other *HOX* genes. This finding suggests that PRKX overexpression may also modulate *HOX* gene mRNA levels through the upregulation of *SHOX*.

Although we obtained interesting results, we cannot directly correlate PRKX effects on cell migration, EMT induction, and *HOX* gene expression to MRKH syndrome determination. VK2 E6/E7 are terminally differentiated epithelial cells, and what we demonstrated using this in vitro model could not happen the same during development. Indeed, inducible in vivo models should be used to investigate the role of PRKX in embryonal tissue morphogenesis and adult tissues homeostasis. However, despite the need for further studies, we laid the basis to speculate that a general dysregulation of *PRKX* expression might be one of the possible causes of MRKH syndrome.

## 5. Conclusions

In conclusion, our work suggests drawing attention to gene expression dysregulation when investigating MRKH syndrome aetiopathogenesis. Indeed, only few MRKH cases could be explained by genetic aberrations, while an altered gene expression pattern may justify a higher number of them. By investigating PRKX dysregulation effects on a model cell line, we contributed toward the identification of a possible molecular cause for MRKH syndrome and highlighted that epigenetics and system biology should always be considered while studying the basis of a complex disease.

## Figures and Tables

**Table 1:** Phenotype of MRKH patients included in the graphical illustration of PCA clusters.

Cluster 1 (Purple)		Cluster 2 (Green)		Cluster 3 (Yellow)	
Patients	MRKH Type	Patients	MRKH Type	Patients	MRKH Type
8	I	7	I	25	II
10	II	9	I	26	I
12	II	11	I	27	I
13	II	14	II	39	II
15	I	19	II	40	I
16	I	20	II		
17	I	22	II		
18	II	29	II		
21	I	31	II		
23	I	33	II		
24	II	35	I		
28	I				
32	I				
34	II				
36	I				
37	I				
41	I				
42	I				

## Data Availability

The data that support the findings of this study are available from the corresponding author upon reasonable request.

## Supplementary Materials

The following is available online at https://www.mdpi.com/article/10.3390/biology10060450/s1, Figure S1: Uncropped Western Blot Images.

## Appendix A

**Principal Component Analysis (PCA) statistics**

PCA performed on RT-qPCR data from MRKH patients by using XLSTAT package for Microsoft Excel.
