# Supplementary material for: Altered Expression of Candidate Genes in Mayer–Rokitansky–Küster–Hauser Syndrome May Influence Vaginal Keratinocytes Biology: A Focus on Protein Kinase X
Source: Biology (Basel). 2021 May 21;10(6):450. doi: 10.3390/biology10060450 (PMC8223793; doi:10.3390/biology10060450)

FIGURE S1

Merges (hybridization + standard) of uncropped blots showed in the final manuscript. Protein lysates from the same experiment were run in different lanes and probed with specific antibodies.

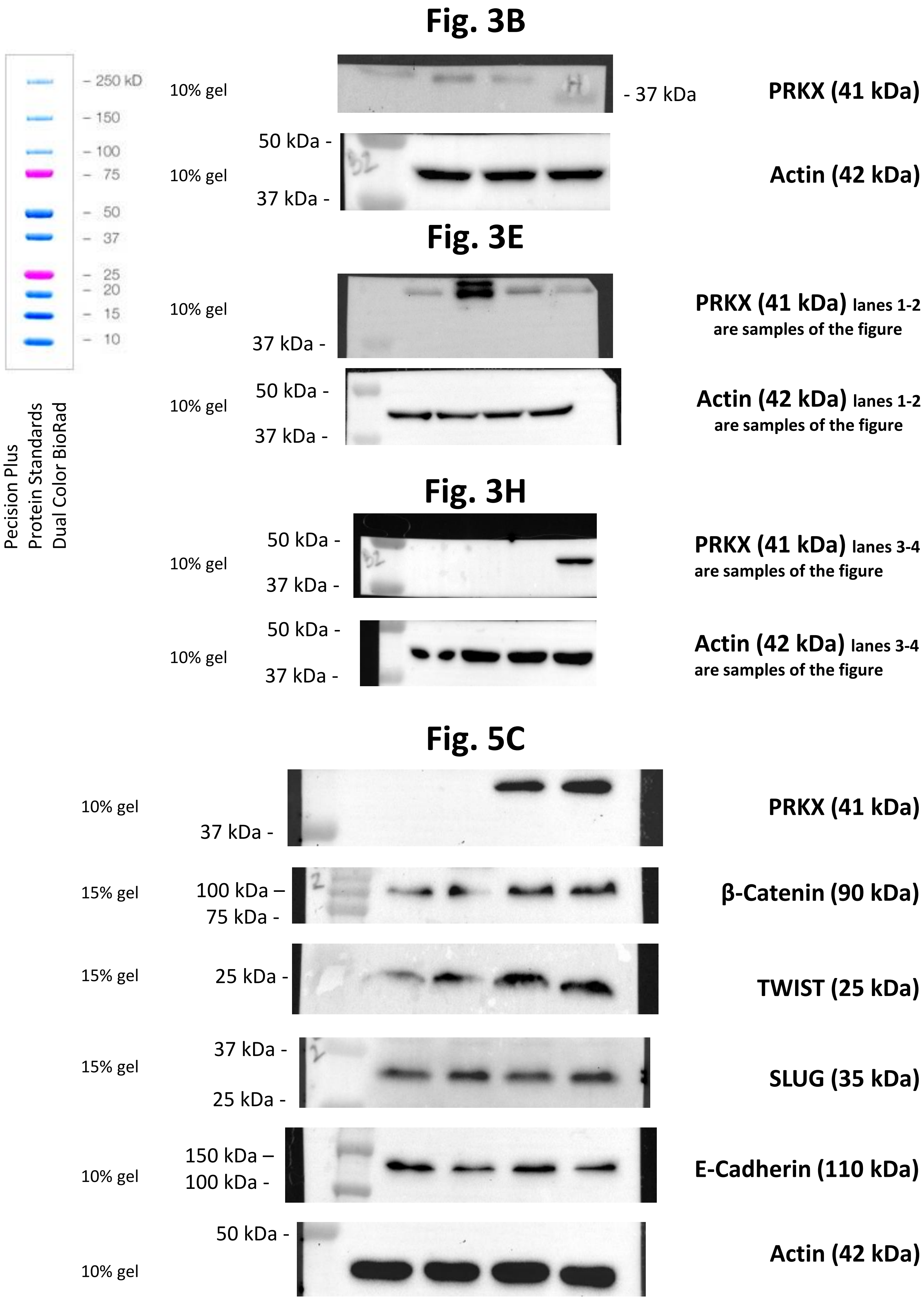

Supplement: Supplementary file 1 [file biology-10-00450-s001.zip › biology-1229186-supplementary.pdf]
